# Mapping health literacy challenges among COPD caregivers: a scoping review

**DOI:** 10.3389/fpubh.2026.1846586

**Published:** 2026-06-19

**Authors:** Tingxu Zhou, Yuyang Wei, Yawen Li, Kun Zhang, Xing Liu, Yanyun Wang, Linping Shang

**Affiliations:** 1 School of Nursing, Shanxi Medical University, Taiyuan, China; 2 The Fifth People's Hospital of Chengdu, Chengdu, China; 3 First Hospital of Shanxi Medical University, Shanxi Medical University, Taiyuan, China

**Keywords:** caregivers, chronic obstructive pulmonary disease, educational needs, health literacy, learning styles

## Abstract

**Objective:**

This study is the first scoping review specifically focused on the health literacy of caregivers of patients with chronic obstructive pulmonary disease (COPD). It aims to summarize the current state of research in this field and provide guidance for the implementation of targeted health education and care support in clinical settings.

**Methods:**

Guided by the scope review methodology, the system searched 10 Chinese and English databases, including PubMed, Web of Science, the Cochrane Library, CINAHL, Embase, Scopus, CNKI, Wanfang Database, VIP Database, and the Chinese Biomedical Literature Database. The search period spanned from the inception of each database to March 16, 2026. Two researchers independently screened, summarized, and extracted data from the search results.

**Results:**

A total of 7 studies were ultimately included, covering 4 countries: China, South Africa, the Netherlands, and the United States. The overall health literacy of caregivers of patients with COPD was found to be relatively low. The main influencing factors included sociodemographic factors, caregiving-related factors, patient-related factors, medical information and education, and the caregivers' physical and psychological factors. Assessment tools lack caregiver-specific instruments, and caregiver education focuses primarily on practical caregiving skills, with learning styles dominated by auditory and literacy-based approaches.

**Conclusion:**

The health literacy levels of COPD caregivers are generally low and influenced by multiple factors, creating an urgent need to develop caregiver-specific health literacy assessment tools. Clinicians should design personalized health education models based on caregivers' needs and learning preferences. Moving forward, high-quality intervention studies are needed to improve caregivers' health literacy and the quality of care, thereby enhancing patient outcomes.

## Introduction

Chronic obstructive pulmonary disease (COPD) is a common chronic airway disease characterized primarily by persistent airflow limitation, shortness of breath, and declining lung function. It is a chronic condition marked by a protracted course and recurrent episodes. Approximately 384 million people worldwide have COPD, with a prevalence rate as high as 11.7% (1). As the disease progresses, patients experience a decline in health and mobility, along with an increase in complications, and become dependent on family caregivers for long-term care and disease management support (2). Caregivers play an indispensable role in disease management and improving health outcomes for patients, and their health literacy is a key factor influencing the effectiveness of patient care (3). Simonds (4) first introduced the concept of health literacy in 1974, defining it as an individual's overall ability to access and understand health information and make health-related decisions based on that information. Nutbeam (5) categorizes it into three levels: functional, interactive, and critical. The World Health Organization (WHO) (6) further emphasizes that health literacy is a key determinant in improving health outcomes and reducing the burden of disease. Caregiver health literacy specifically refers to a caregiver's ability to identify care needs that arise while caring for a patient, and—in response to those needs—to actively seek out, understand, and apply relevant information and services, as well as to assist the patient or make decisions on their behalf, thereby maintaining or promoting the patient's health (7). Inadequate health literacy among caregivers can lead to improper care practices, inaccurate symptom recognition, poor adherence to disease management, increased frequency of acute exacerbations and risk of hospitalization, and higher healthcare costs, thereby exacerbating the global burden of COPD management (8). Current research, both domestically and internationally, has largely focused on the health literacy of COPD patients themselves. Studies specifically targeting the health literacy of caregivers remain limited in number and vary widely in scope, and no unified research framework has yet been established (9). At the same time, existing health literacy assessment tools are primarily designed for patients and do not align with the role characteristics and practical needs of caregivers. Furthermore, relevant health education models do not account for caregivers' learning patterns, making it difficult to meet the requirements for delivering precise clinical care support (10). Given that research in this field is still in its early stages and faces challenges such as fragmented research topics and insufficient evidence from intervention studies, this study will employ an exploratory review rather than a systematic review. An exploratory review is better suited to comprehensively mapping the current state of research in the field, identifying existing knowledge gaps, and outlining the overall research landscape (11). Based on this, this study employs Arksey's (12) scoping review framework as its methodological guide, integrating relevant theories from Nutbeam and the WHO on health literacy. It systematically reviews the current status, influencing factors, assessment tools, educational needs, and learning characteristics of health literacy among COPD caregivers. The aim is to provide a theoretical foundation and practical guidance for clinicians to develop a targeted health education system for caregivers, optimize care support strategies, and alleviate the burden of comprehensive COPD management.

## Additional requirements

### Define the research question

#### Main research questions

- What is the level of health literacy among caregivers of patients with COPD?
- What factors influence the health literacy of caregivers of patients with COPD?
- What tools are available for assessing the health literacy of caregivers of patients with COPD?
- What are the learning styles and educational needs of caregivers of patients with COPD?

### Inclusion and exclusion criteria

Inclusion and exclusion criteria were established based on the PCC principles. Inclusion criteria: (1) Participants (P) must be family caregivers of patients with COPD, or the study must include both patients and caregivers; (2) Concepts (C) must relate to topics such as health literacy, disease knowledge, and caregiving knowledge; (3) Contexts (C) must involve caregiving settings such as the community or the home. Exclusion criteria: Not in Chinese or English; Full text unavailable; Reviews, systematic reviews, conference papers, research proposals, etc.; Duplicate publications.

### Search strategy

We searched PubMed, Web of Science, the Cochrane Library, CINAHL, Embase, Scopus, CNKI, Wanfang Database, VIP Database, and the Chinese Biomedical Literature Database. The search period spanned from the inception of each database to March 16, 2026. We used a combination of subject headings and free-text terms for the search. Taking PubMed as an example for English-language databases, the search query was:(((((((([Pulmonary Disease, Chronic Obstructive (MeSH Terms)] OR [Chronic Obstructive Pulmonary Diseases (Title/Abstract)] OR [COPD (Title/Abstract))] OR (Chronic Obstructive Lung Disease [Title/Abstract])) OR (Chronic Obstructive Pulmonary Disease [Title/Abstract])) OR (Chronic Disease[Title/Abstract])) OR (Chronic Diseases[Title/Abstract])) OR (Disease, Chronic [Title/Abstract])) OR (Chronic Illness[Title/Abstract])) OR (Chronic Illnesses [Title/Abstract]) AND((Health Literacy[MeSH Terms]) OR (Health Literacy Questionnaire[Title/Abstract])) OR (Health Literacy Scale[Title/Abstract])AND(((((((((((Pulmonary Disease, Chronic Obstructive[MeSH Terms]) OR (Chronic Obstructive Pulmonary Diseases[Title/Abstract])) OR (COPD[Title/Abstract])) OR (Chronic Obstructive Lung Disease[Title/Abstract])) OR (Chronic Obstructive Pulmonary Disease[Title/Abstract])) OR (Chronic Disease [Title/ Abstract])) OR (Chronic Diseases[Title/Abstract])) OR (Disease, Chronic [Title/Abstract])) OR (Chronic Illness[Title/Abstract])) OR (Chronic Illnesses[Title/Abstract])) AND (((Health Literacy[MeSH Terms]) OR (Health Literacy Questionnaire[Title/Abstract])) OR (Health Literacy Scale[Title/Abstract]))) AND (((((caregivers[MeSH Terms]) OR (Caregiving[Title/Abstract])) OR (informal caregivers[Title/Abstract])) OR (Family caregivers[Title/Abstract])) OR (Primary Caregiver [Title/ Abstract])).

### Literature screening and data extraction

After importing the references into EndNote and removing duplicates, two researchers independently screened the references. In cases of disagreement, they consulted a third researcher to determine whether to include the reference. Data extracted included the authors, publication date, country, study population, study design, sample size, and key research findings.

## Results

### Results of the literature screening

**Figure 1:**
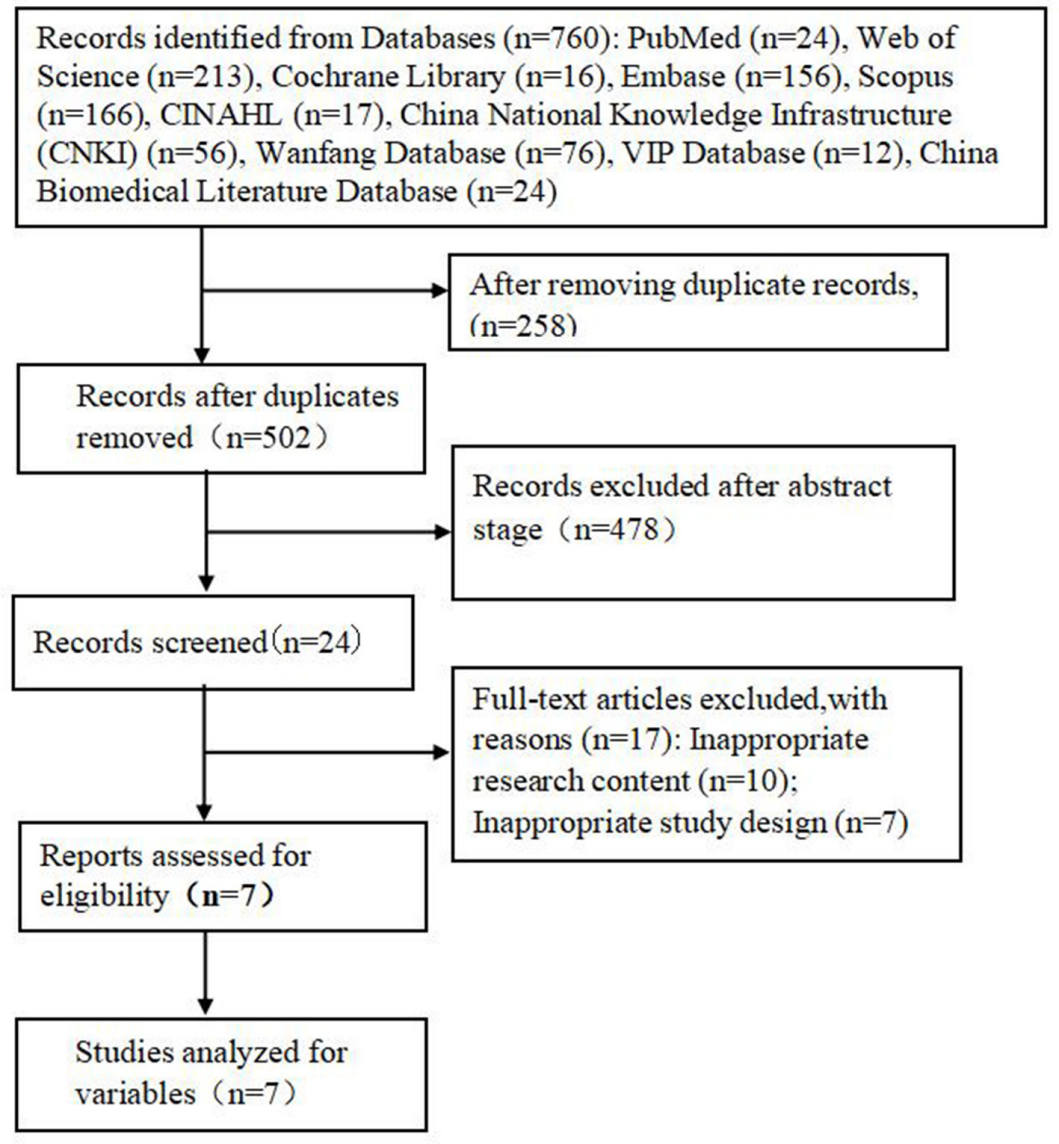
Flow chart of literature screening.

An initial search yielded a total of 760 articles; after removing duplicates, 502 remained. After reviewing the titles and abstracts, 478 articles were excluded; following a secondary screening of the full texts, 17 articles were further excluded, resulting in the final inclusion of 7 articles. The literature screening process is shown in Figure 1.

### Basic characteristics of the included literature

Of the 7 studies included in this review, the publication dates ranged from 2019 to 2026. Two were in Chinese (13, 14), and five were in English (15–19), four were from China (13, 14, 17, 19), one from South Africa, one from the Netherlands (18), and one from the United States (15). Study designs included cross-sectional studies (*n* = 5) (13, 14, 17–19), qualitative studies (*n* = 1) (16) and prospective cohort studies (*n* = 1) (15). The basic characteristics of the included studies are shown in Table 1.

**Table 1:** Characteristics of the included studies (*n* = 7).

References	Country	Aims	Study design	Study population	Sample size	Application method	Outcomes	Interventions/ future applications	Factors
Gwyther et al. (16)	South Africa	Exploring palliative care needs and gaps among COPD patients, caregivers, and healthcare providers in primary care settings in South Africa	Qualitative research	Family caregivers for COPD patients	19	–	Caregivers have low health literacy regarding the diagnosis, progression, and palliative care of COPD	None/improve doctor-patient communication and streamline information sharing	Educational level, income, psychological factors, information dissemination, quality of communication, and access to healthcare resources
Yueling et al. (14)	China	Understanding the current state of health literacy among COPD patients and their family caregivers, and its influencing factors	Cross-sectional study	People with COPD and their caregivers	238	COPD-Q	The health literacy score rate among caregivers was 46.4%, placing it in the lower-middle range.	None/simultaneous health education for patients and caregivers	Educational level, income, health status, ability to provide care, age, marital status, adherence to oxygen therapy, place of residence
Wang N et al. (19)	China	Demographic and educational factors associated with the level of disease knowledge among family caregivers of patients with COPD	Cross-sectional study	Primary caregivers for people with COPD	726	BCKQ	47.1% of caregivers did not meet the passing standard for disease knowledge	None/conducting specialized health education on COPD	Educational level, age, marital status, health education, and psychological factors
Muijsenberg et al. (18)	Netherlnds	Identifying the educational needs, learning styles, and psychological characteristics of people with COPD and their caregivers	Cross-sectional study	People with COPD and their primary caregivers	67	LINQ, HLS-EU-Q16	Caregivers have greater information needs than patients and primarily learn through auditory and reading/writing methods.	None/offers multi-modal personalized education	Psychological factors
Muellers et al. (15)	United States	Exploring the mitigating effect of caregiver support on medication adherence among COPD patients with low health literacy	Prospective cohort study	People with COPD and their caregivers	97	S-TOFHLA, MARS	Caregivers have low health literacy, which prevents them from improving patients' medication adherence	None/interventions targeting caregivers with low health literacy	Educational attainment, income, psychological factors, race
Ma B et al. (17)	China	Analysis of subtypes of self-care contributions among caregivers of COPD patients and associated factors	Cross-sectional study	People with COPD and their caregivers	275	COPD-Q, MS, CSMCS, CC-SCCII	Caregivers' health literacy is generally low and unevenly distributed	None/tiered approach to enhancing the competence of caregivers with different roles	Educational level, social support, caregiving capacity, psychological factors, health education, patient-related factors
Wenjie and Yueling. (13)	China	Exploring the relationship between caregivers' health literacy and caregiving abilities and patients' pulmonary rehabilitation	Cross-sectional study	People with COPD and their caregivers	238	COPD-Q, ?=FCTI	There is a positive correlation between caregivers' health literacy and caregiving ability and patients' pulmonary rehabilitation outcomes.	None/enhancing care capabilities and patient recovery through improved health literacy	Educational level, income, health status, adherence to oxygen therapy, caregiving capacity, patient-related factors, place of residence

COPD-Q refers to the COPD Health Literacy Scale; BCKQ refers to the Bristol COPD Knowledge Questionnaire; LINQ refers to the Lung Disease Information Needs Questionnaire; HLS-EU-Q16 refers to the European Health Literacy Scale (Short Version); S-TOFHLA refers to the Short Version of the Functional Health Literacy Assessment; MARS refers to the Medication Adherence Scale; MS refers to the Intimacy Scale; CSMCS refers to the Caregiver Self-Care Management Confidence Scale; CC-SCCII refers to the Chronic Disease Caregiver Self-Care Contribution Scale; FCTI refers to the Chinese Version of the Family Caregiver Competence Index.

### Health literacy levels among caregivers of patients with COPD

Seven studies reported on caregivers' health literacy levels; the overall findings indicate that health literacy among caregivers both domestically and internationally remains generally low. Two studies (13, 14) showed that caregivers' health literacy scores averaged 46.4%, indicating a lower-middle level of health literacy; only four items had a correct response rate exceeding 60%, suggesting poor mastery of core knowledge. One study (16) conducted in-depth interviews with 19 family caregivers of people with COPD and found that these caregivers had low health literacy regarding COPD diagnosis, disease progression, and palliative care. One study (19) found that nearly half of caregivers (47.1%) did not meet the passing standard for knowledge of underlying medical conditions, indicating insufficient health literacy. One study (17) shows that caregivers' health literacy is generally low and unevenly distributed, and that the literacy levels of low-contribution caregivers urgently need to be improved.

### Factors influencing health literacy among caregivers of patients with COPD

This study identified five major themes encompassing 16 influencing factors, including sociodemographic factors, care-related factors, patient-related factors, medical information and education factors, and caregiver-related factors. Socio-demographic factors: age (13, 14, 19), educational attainment (13–17, 19), marital status (14, 19), income (13–16), place of residence (13, 14) and race (15). Care-related factors: whether patients have received COPD-related health education (16, 17, 19), caregiving capacity (13, 17), adherence to home oxygen therapy (13, 14) and caregiving support (16, 17). Patient-related factors: The patient's physical condition (13, 14, 17). Healthcare information and educational factors (16): information dissemination, quality of communication, and healthcare resources. Caregiver-related factors: physical health (13, 14), and mental health (16–18).

### Assessment tools for health literacy among caregivers of patients with COPD

The included studies covered a total of 10 assessment tools, five of which were health literacy assessment tools for caregivers: the Chronic Obstructive Pulmonary Disease Knowledge Questionnaire (COPD-Q) (20), has a Cronbach's alpha coefficient of 0.72 and a test-retest reliability of 0.90; the content validity ratios for individual items range from 0.40 to 0.91. Currently, while it has been translated and adopted in many countries, it is designed exclusively for patients and does not address the specific needs of caregivers in their caregiving roles. It assesses only disease-related knowledge and lacks dimensions related to interactive and critical health literacy. The Bristol COPD Knowledge Questionnaire (BCKQ) (21), has good internal consistency, with a Cronbach's alpha coefficient of 0.73 and a test-retest reliability of 0.71. As an assessment tool for evaluating patients' knowledge of COPD, it contains numerous items and is burdensome to complete; it does not take into account the learning characteristics of caregivers; it covers only disease knowledge without addressing caregiving skills or the ability to apply health literacy; and it lacks re-validation of its reliability and validity specifically for the caregiver population. The Lung Information Needs Questionnaire (LINQ) (22) has a Cronbach's alpha coefficient of 0.73 and a test-retest reliability of 0.89. It assesses, from the patient's perspective, the information needed to fully understand lung diseases and maximize self-management skills. However, it does not align with the specific information needs of caregivers; it merely identifies gaps in information needs without directly assessing health literacy levels. The European Health Literacy Survey Questionnaire 16 (HLS-EU-Q16) (23) has a Cronbach's alpha coefficient of 0.89. This concise questionnaire possesses adequate psychometric properties for assessing participants' health literacy levels. However, the absence of a COPD-specific assessment tool means that it cannot reflect caregivers' competence in core care tasks such as oxygen therapy, medication administration, and management of acute exacerbations, and it lacks caregiver-specific validation and validity testing. The Short Test of Functional Health Literacy in Adults (S-TOFHLA) (24) has a Cronbach's alpha coefficient of 0.97 and a test-retest reliability of 0.89. It is concise and efficient, making it suitable for rapid clinical screening and large-scale epidemiological surveys of health literacy levels. However, assessing only functional health literacy overlooks interactive and critical literacy, lacks caregiver-specific items, and does not incorporate the context of home care for COPD.

### Learning styles and educational needs of caregivers for patients with COPD

Caregivers have significantly higher overall information needs than patients, with the top three areas being self-management, exercise, and diet. Additionally, their need for medication-related information is significantly higher than that of patients. These patterns indicate that, because they bear the responsibility of providing care, caregivers are more focused on practical caregiving skills and details regarding medication use, rather than merely basic knowledge about the disease. Caregivers prefer auditory (49%) and written (36%) learning styles; auditory learning is ideal for real-time explanations and Q&A sessions, while written learning is best for retaining care guidelines that can be reviewed repeatedly. More than half of caregivers (50.7%) are multimodal learners (18).

## Discussion

### The overall level of health literacy among COPD caregivers is generally low, and there are significant knowledge gaps

The results of this study indicate that the health literacy of family caregivers of COPD patients is generally at a lower-middle level, with insufficient understanding of disease-related knowledge and low awareness of relevant information. Consistent with findings from studies on caregivers of patients with other chronic conditions such as diabetes (25), heart failure (26), and stroke (27), this suggests that insufficient health literacy among caregivers is a common issue in the field of chronic disease care. However, COPD is characterized by progressive disease progression, recurrent acute exacerbations, and long-term home oxygen therapy. Consequently, the health literacy gap is more concentrated in practical areas such as recognizing acute exacerbations, adhering to standard oxygen therapy protocols, using inhalers, and managing emergencies, reflecting distinct differences in care scenarios compared to caregivers of other chronic diseases (28). As a progressive and irreversible chronic respiratory disease, COPD requires long-term care, involves complex and variable symptoms, and demands a high degree of self-management. However, caregivers are often family members rather than professionals, and they lack a systematic understanding of the disease (28). At the same time, most caregivers juggle multiple responsibilities—including daily care, emotional support, and disease management—while facing limited energy and relying on a single source of information, which further exacerbates their lack of health literacy (29). From a holistic perspective of family care, family care is not limited to the acquisition of static knowledge about diseases; rather, it requires caregivers to dynamically respond to various unexpected care situations, such as acute exacerbations, symptom fluctuations, and medication errors (30). Existing research has largely focused on static education regarding the basic theoretical knowledge of COPD, lacking practical training on how to handle dynamic care emergencies. This approach not only overlooks the core role of caregivers in coordinating multiple healthcare resources and bridging the gap between hospital-based treatment and home care, but also fails to establish targeted, supportive training programs for caregivers (31).

As can be seen, the low health literacy among COPD caregivers stems not only from the nature of the disease itself, the unique caregiving context, and the caregivers' own lack of knowledge, but also from the fact that the current health education and training systems fail to meet their actual caregiving needs. These systems lack training on coordinating medical resources and facilitating seamless referrals between hospitals and community settings, lack immediate support plans for handling unexpected caregiving incidents, and fail to design targeted interventions based on clinical realities. It is worth noting that even when patients are accompanied by a caregiver to medical appointments and the caregiver participates in medical discussions, the issue of low health literacy among patients persists (15), it is suggested that interventions targeting patient populations with low health literacy should explore ways to involve both patients and their caregivers in health education, as well as whether it is possible to leverage the strengths of the patient-caregiver relationship to mitigate the adverse effects of low health literacy. Future research could focus on addressing the above issues by providing timely support and innovative interventions for caregivers: (1) Digital health support: Develop resources tailored to auditory and literacy-based learning styles, such as short videos, voice-guided instructions, and mini-programs featuring first-aid flowcharts. (2) Culturally adapted programs: Provide large-print, simplified, and pictorial tools for caregivers with low educational attainment, those in rural areas, and older adults. (3) Dual-track education: Integrate caregivers into the comprehensive management of COPD by implementing synchronized health education for both patients and caregivers during outpatient visits, discharge instructions, and home follow-ups.

### The factors influencing health literacy among COPD caregivers are multidimensional and highly amenable to intervention

The results of this study indicate that the health literacy of COPD caregivers is influenced by five major factors: sociodemographic factors, caregiving-related factors, patient-related factors, healthcare system factors, and the caregivers' physical and mental health. These factors interact to form a complex network of influences, and most of them are amenable to intervention. Sociodemographic factors: Age, educational attainment, marital status, income, place of residence, and race are all important factors influencing caregivers' health literacy. Among these, educational attainment is a significant factor; the higher the level of education, the higher the level of health literacy (13–17, 19, 32), at the same time, caregivers who live in urban areas, have higher incomes, are married, and are of middle age tend to have higher levels of health literacy, while caregivers from ethnic minority groups tend to have relatively lower levels of health literacy, This reflects how external social conditions—such as socioeconomic status, living environment, and family support—play a moderating role in caregivers' access to health information, knowledge acquisition, and the improvement of their health literacy. Caregiving-related factors: Whether caregivers receive COPD-related health education, their caregiving capabilities, adherence to home oxygen therapy protocols, and the level of caregiving support directly influence their health literacy. Caregivers who have received specialized health education, possess strong caregiving skills, follow proper procedures for home oxygen therapy, and receive adequate caregiving support tend to have higher levels of health literacy (16, 17, 19), Systematic, targeted COPD-related health education, care training, and support can enhance caregivers' self-efficacy in providing care. This strengthens their sense of competence and confidence in recognizing changes in the patient's condition, assessing symptom risks, managing unexpected issues, and handling daily care, enabling them to implement standardized care practices more proactively and consistently (33). At the same time, such training can effectively alleviate caregivers' own care-related stress, anxiety, and physical and mental burdens, reduce caregiver burnout, and improve the continuity and stability of care. This may be attributed to the fact that formal care education not only compensates for knowledge gaps in experiential care but also promotes the transformation of health literacy from passive knowledge to active practice by boosting care confidence, alleviating psychological stress, and optimizing the care experience (34). Patient-related factors: The patient's physical condition is an external factor (13, 14, 17), the severity of the patient's condition, the number of acute exacerbations within 1 year, comorbidities, and frequency of hospitalizations are all negatively correlated with the caregiver's health literacy. The poorer the patient's physical condition and the more frequent the fluctuations in their condition, the more likely caregivers are to focus on emergency response rather than acquiring systematic knowledge, thereby lowering their health literacy levels (35, 36). As a progressive chronic disease, COPD involves significant differences in care needs across its various stages. However, only a limited number of studies (15–17) have examined the association between disease severity and caregivers' health literacy. Existing research (13, 14, 18, 19) generally lacks stratified analysis based on disease severity and does not provide tailored training according to the stage of the disease, resulting in a mismatch between care support and actual needs. For patients with mild COPD, care primarily involves daily symptom management, medication adherence, and pulmonary rehabilitation; the caregiver's core task is to assist in maintaining a stable condition. Patients with moderate-to-severe COPD often have multiple comorbidities and an increased risk of acute exacerbations, placing a higher care burden on caregivers. These caregivers need to master skills in symptom monitoring, dyspnea control, emergency response, and cross-institutional medical coordination (37). It is evident that as the severity of the disease increases and the disease progresses to later stages, the care knowledge and skills required of caregivers become more complex and closely related to emergency scenarios. The lack of tiered, precise care support tailored to the specific stage of the disease is precisely the key area requiring improvement in future clinical practice. Medical information and educational factors: Information dissemination, quality of communication, and medical resources are environmental factors that influence caregivers' health literacy (16). Poor communication between doctors and patients, fragmented health information, insufficient medical resources, unstable drug supplies, and poor continuity of care can all lead to caregivers having insufficient knowledge about diseases, which in turn contributes to low health literacy. Caregiver-related factors: The caregiver's physical and mental health and psychological state are often overlooked yet crucial internal factors (16, 17). When caregivers themselves suffer from chronic illnesses or physical exhaustion, their ability to acquire knowledge and master skills is directly impaired. Meanwhile, negative psychological factors such as anxiety, worry, and sleep disorders, coupled with low self-efficacy in caregiving, reduce their motivation to seek information and their learning efficiency, creating a vicious cycle of low health literacy and high caregiving burden (38–40).

Among the aforementioned factors, aside from demographic characteristics such as age and educational attainment, health education, caregiving support, improved communication, and community services are all modifiable variables. These provide a direction for developing targeted improvement strategies and demonstrate that caregivers' health literacy is not fixed but can be improved through systematic interventions (41).

### Tools for assessing health literacy among COPD caregivers primarily consist of disease-specific scales; tools specifically designed for caregivers have yet to be developed

Current assessment tools for health literacy among caregivers of people with COPD can be divided into two categories: general health literacy scales and disease-specific scales, each with its own advantages and disadvantages and differing applications.

Generalized scales, such as the S-TOFHLA (Short Test of Functional Health Literacy in Adults), which is derived from the Baker Simplified Test of Functional Health Literacy in Adults (TOFHLA) (24). As well as Sørensen K's adaptation of the European Health Literacy Survey Questionnaire (HLS-EU-Q) into the HLS-EU-Q16 (European Health Literacy Survey Questionnaire16, HLS-EU-Q16), among others (23). While these scales have broad applicability and demonstrate reliable validity and reliability, they lack specificity to the COPD care setting and cannot accurately reflect caregivers' knowledge and skill levels in core care tasks such as symptom monitoring, medication management, and managing acute exacerbations. Among disease-specific scales, the Chronic Obstructive Pulmonary Disease Health Literacy Questionnaire (COPD-Q) (20) is the most widely used. This scale is primarily designed to assess patients' knowledge of health literacy and includes 13 items covering COPD symptoms, risk factors, prevention, and other topics. It is concise and quick to complete, making it suitable for rapid screening in clinical and community settings; it was the most commonly used tool in the studies included in this review. In addition, the Bristol COPD Knowledge Questionnaire (BCKQ) (21) includes expanded content such as pulmonary rehabilitation, oxygen therapy, and vaccinations, making it suitable for detailed assessment; however, it contains a large number of items and is time-consuming to complete.

Existing assessment tools have significant limitations: First, they do not utilize scales specifically designed to assess caregivers' health literacy; most simply adapt patient-specific scales without fully considering caregivers' information needs, caregiving roles, and learning characteristics. Second, there is a lack of comprehensive health literacy assessment tools that integrate functional, interactive, and critical components, making it difficult to fully capture the multidimensional nature of health literacy. Compared with other chronic diseases, specific health literacy scales for caregivers have been gradually developed in fields such as stroke (42), cancer (7), and heart failure (43), whereas in the field of COPD, scales continue to be developed primarily for patients, lagging behind the research standards of comparable chronic diseases. Future research should focus on developing health literacy assessment tools for COPD caregivers specifically tailored to this population, thereby providing reliable tools for precise clinical assessment and intervention.

### The educational needs of COPD caregivers are particularly pronounced and highly individualized, with diverse preferences regarding learning styles

Included studies indicate (18, 44) that caregivers of individuals with COPD have unmet educational needs, with a focus on practical content related to disease management; furthermore, their learning styles are diverse, necessitating the implementation of personalized educational interventions. In terms of educational needs, caregivers' overall information needs are significantly higher than those of patients. Their core needs are concentrated in the areas of self-management, exercise, and diet, and their demand for medication-related information is also significantly higher. However, their demand for disease-related knowledge and smoking-related information does not differ significantly from that of patients. Their needs are more oriented toward practical caregiving skills rather than purely theoretical knowledge of the disease, which aligns with their role in providing care and assisting with disease management (45). In terms of learning styles, caregivers primarily prefer auditory and reading/writing-based approaches; nearly half of them favor multimodal learning, while also exhibiting kinesthetic learning needs, which aligns with the learning patterns of caregivers for patients with chronic conditions. Additionally, caregivers tend to exhibit “fighter” personality traits and prefer direct, concise, and goal-oriented communication, suggesting that educational content should be concise, clear, and focused on key points.

It is worth noting that current clinical education tends to be patient-centered, neglecting the simultaneous education of caregivers, and often relies on a one-sided, lecture-style approach that fails to account for individual learning styles and psychological characteristics, resulting in suboptimal educational outcomes (46). Therefore, future interventions must be guided by the needs of caregivers and adopt multi-modal, personalized, and practical educational programs that also reach vulnerable populations with low health literacy, thereby ensuring educational equity and maximizing effectiveness. Furthermore, and more importantly, training on health literacy for caregivers and standardized assessments should be formally incorporated into COPD clinical pathways and comprehensive management at the institutional level, making them essential components of inpatient education, discharge instructions, and long-term management, thereby ensuring the systematic and sustained nature of caregiver health education through policy measures.

## Limitations and future directions

This study is the first in the field of COPD to innovatively focus on caregivers' health literacy, filling a gap in previous research that tended to focus on patients' own health literacy while overlooking the central role of caregivers, and providing a new direction for research on COPD care management. A key limitation of this study is that it included only seven studies. The included studies were primarily from China, Europe, and the United States, resulting in an uneven geographical distribution. The lack of data from different countries and cultural backgrounds limits the representativeness of the findings. Second, this study is a scoping review that primarily aims to systematically organize and describe the current state of research. It does not conduct rigorous quality assessments, evidence grading, or in-depth critical synthesis of the included literature; therefore, the strength of the evidence supporting the conclusions is limited. Some studies have simple designs, making it difficult to conduct in-depth methodological comparisons and meta-analyses. Third, most existing studies rely on patient-based questionnaires rather than health literacy assessment tools specifically designed for COPD caregivers, making it impossible to accurately align the assessment with the caregivers' specific roles and needs, and resulting in biased findings.

Future research should focus on developing and validating a health literacy assessment tool specifically designed for caregivers. Based on the caregiving responsibilities, information needs, and learning characteristics of COPD caregivers, a specialized scale that combines reliability, validity, and clinical applicability should be developed. This process should include item selection, reliability and validity testing, and validation of clinical application to address the core issue of the current lack of assessment tools. Conduct large-scale, multicenter longitudinal studies to track long-term trends in the health literacy of COPD caregivers, thereby providing a basis for phased interventions. Develop personalized caregiver training and digital intervention models, and create standardized training modules tailored to caregivers' learning styles—primarily auditory and literacy-based. Explore digital intervention measures such as short videos, mini-programs, and online Q&A sessions, while ensuring these are adaptable to caregivers from diverse cultural backgrounds and educational levels to enhance the accessibility of these interventions. Conduct a comparative study on the health literacy of caregivers for chronic diseases such as COPD, diabetes, and cardiovascular and cerebrovascular diseases; identify the unique characteristics and common patterns of health literacy among COPD caregivers; and refine the research framework for the health literacy of caregivers for chronic diseases. Promote the incorporation of caregiver health literacy into clinical management guidelines for COPD, establish a model for simultaneous health education for patients and caregivers, and achieve a synergistic improvement in both the quality of care and patient outcomes.

## Summary

This study indicates that the overall health literacy of family caregivers of COPD patients is low, influenced by multiple factors including sociodemographic characteristics, caregiving factors, patient condition, medical information, and the caregivers' physical and mental wellbeing. Most existing assessment tools are adaptations of patient-specific scales and lack versions designed specifically for caregivers. caregivers have high information needs and primarily rely on auditory and literacy-based learning, making them suitable for multimodal, personalized education. It is recommended that clinicians expedite the development of health literacy assessment tools specifically designed for caregivers, establish a needs-oriented, learning-style-adapted precision health education model, and integrate both patients and caregivers into the comprehensive management of COPD. However, the included literature in this study exhibits uneven geographical distribution, and no quality assessment or evidence grading was conducted, resulting in limited evidence strength. Future research should involve large-sample, multicenter intervention studies to refine the assessment and intervention systems and enhance clinical applicability.

## Data Availability

The original contributions presented in the study are included in the article/supplementary material, further inquiries can be directed to the corresponding author.
